# Megaprosthesis Infection by *Bacteroides fragilis* in a Patient With Ewing's Sarcoma: A Case Report and Literature Review

**DOI:** 10.1002/ccr3.70185

**Published:** 2025-02-09

**Authors:** Yoleidys Martínez‐Ysasis, Antonella Francesca Simonetti, Ferran Navarro

**Affiliations:** ^1^ Microbiology Department Hospital de la Santa Creu i Sant Pau Barcelona Spain; ^2^ Infectious Diseases Unit Hospital de la Santa Creu i Sant Pau Barcelona Spain; ^3^ Genetics and Microbiology Department Universitat Autònoma de Barcelona Barcelona Spain; ^4^ Sant Pau Institute of Biomedical Research (IIb Sant Pau) Barcelona Spain

**Keywords:** *Bacteroides fragilis*, Ewing's sarcoma, joint infection, megaprosthesis

## Abstract

*Bacteroides fragilis* , typically linked to intra‐abdominal infections, caused a rare prosthetic infection in a patient with Ewing's sarcoma. Treated with intravenous and oral antibiotics, the patient fully recovered. This case underscores the diverse infections *B. fragilis* can cause and highlights the need for further research.

## 1 Introduction

*Bacteroides fragilis* is an anaerobic, non‐spore‐forming, bile‐resistant, gram‐negative bacillus. It has been identified as a causative agent in various pathologies, particularly polymicrobial intra‐abdominal infections. Presented here is a unique case of a prosthetic infection (megaprosthesis) linked to *B. fragilis* in a patient diagnosed with Ewing's Sarcoma, a circumstance hitherto unreported in the literature.

## 2 Case History/Examination

A 59‐year‐old female patient with a history of hypothyroidism, chronic migraine, and Ewing's Sarcoma of the right acetabulum underwent hip reconstruction with a megaprosthesis in May 2022. Her treatment included preoperative radiotherapy and chemotherapy. However, the continuation of chemotherapy was deemed inadvisable due to her general condition and the elevated risk of complications. She was admitted to a social healthcare center for post‐surgery recovery in July 2022, remaining there until September 2022, when she was referred to the emergency room with a suspected megaprosthesis infection. Empirical treatment with amoxicillin/clavulanic acid was initiated. Upon arrival at the emergency room, the patient underwent a traumatological assessment. Physical examination revealed the presence of erythema, edema, redness, and warmth at the right hip, upper vulvar lip, and lateral side of the ipsilateral thigh. No additional symptoms or signs of sepsis were observed, and the patient denied any preceding trauma.

## 3 Methods

While the patient was in the emergency room, a urine sample was obtained for culture, in accordance with hospital protocol (patient with a urinary catheter), yielding a positive result for *Pseudomonas aeruginosa* . An initial analysis revealed a hemoglobin level of 8.9 g/dL and leukocytes of 7800 g/dL, along with a C‐reactive protein level of 368.8 mg/L. At this juncture, a decision was made to administer a transfusion of 2 units of packed red blood cells. Following the blood transfusion, the anemia showed no signs of worsening. Consequently, the empirical treatment was modified to piperacillin/tazobactam to broaden the antibiotic spectrum.

**FIGURE 1:**
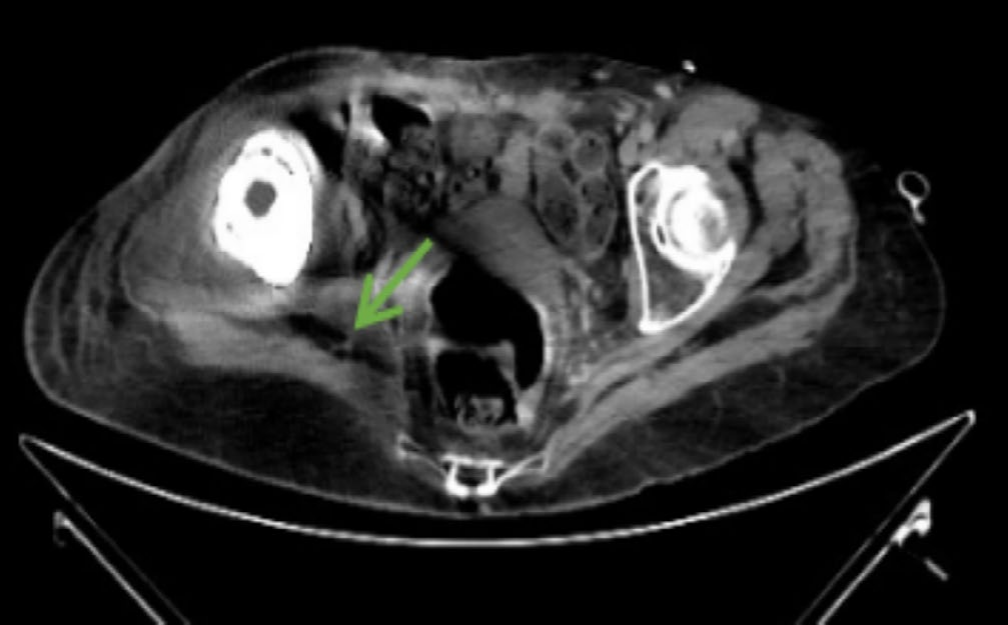
A non‐contrast CT scan showing the presence of air bubbles in the right hemipelvis.

A computerized tomography scan was performed, revealing an abscess in the obturator region and right pelvic floor measuring 5 × 5 × 4 cm. Additionally, small, deep laminar collections were observed in the upper parasagittal region of the right gluteus medius and the lateral costal wall (Figure 1). Based on these findings, the patient was diagnosed with a right hemipelvic megaprosthesis abscess and admitted for further management.

An urgent surgical intervention was carried out, which included debridement of the infected area with implant retention and intraoperative sampling. Samples were collected for subsequent microbiological culture, including a specimen from the proximal groin, a periprosthetic tissue biopsy, and two samples of purulent articular material, which were promptly dispatched to the microbiology service. Following surgical debridement and in response to the suspected infection involving Gram‐positive cocci, vancomycin was incorporated into the empirical treatment regimen and administered intravenously.

Upon receiving the samples, the microbiology service performed a Gram stain, which did not reveal any microorganisms. The joint fluid did not undergo any prior processing such as centrifugation, and blood culture bottles were not utilized. Subsequently, the samples were cultured on appropriate media for aerobic (blood agar and chocolate agar) and anaerobic bacteria (Schaedler agar). Additionally, a liquid enrichment medium (thioglycolate) was used.

**FIGURE 2:**
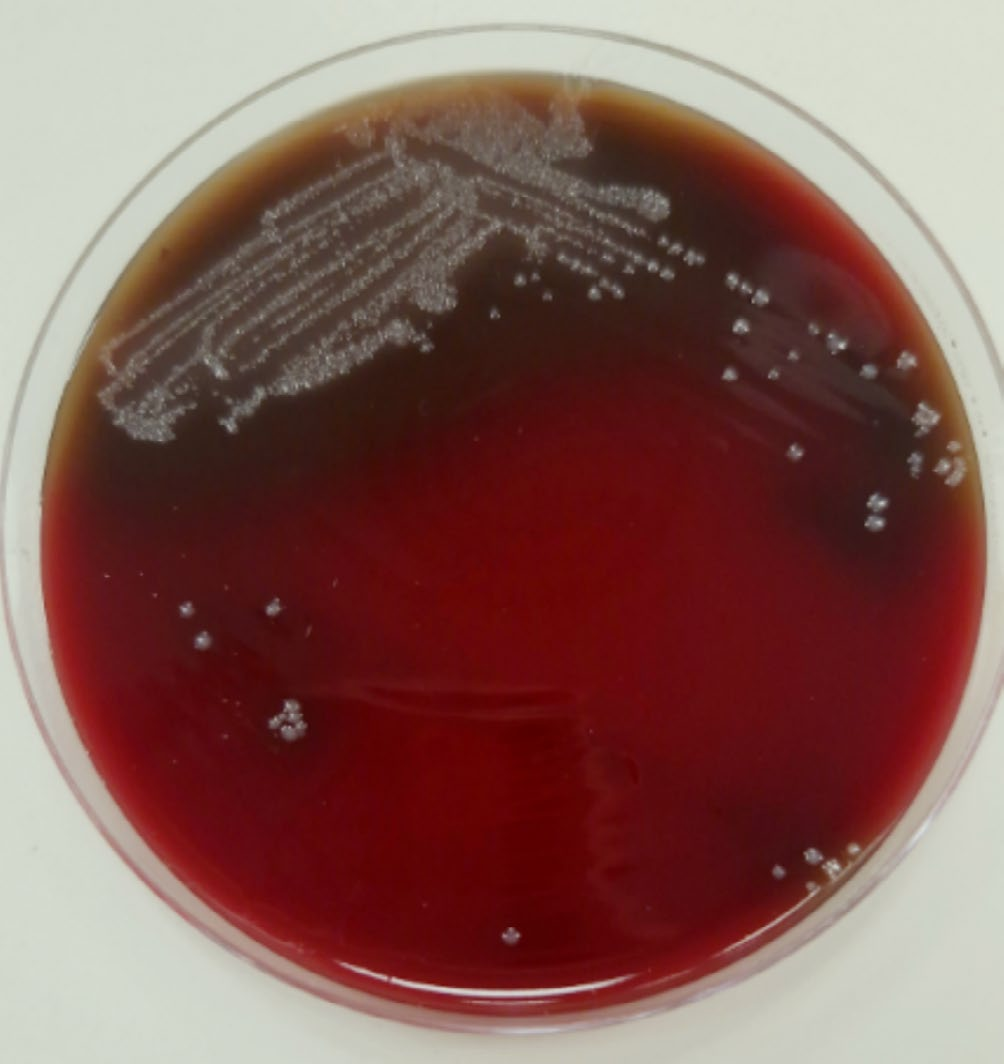
Small, grayish colonies of *B. fragilis* on Schaedler agar.

After 48 h, the bacteriological study of the drained material showed small, grayish colonies on Schaedler agar (Figure 2). *B. fragilis* was isolated in pure culture from all four samples, whereas the aerobic cultures were negative. Blood cultures were not performed.

The identification of the isolated microorganism was confirmed using mass spectrometry (matrix‐assisted laser desorption/ionization time‐of‐flight mass spectrometry [MALDI‐TOF MS] Bruker, Germany), yielding *B. fragilis* with a score of 2.23. Antimicrobial susceptibility testing was performed by the gradient diffusion method (Liofilchem, Italy) in Mueller‐Hinton Fastidious agar (BioMerieux, France).

The isolated *B. fragilis* strain was found to be susceptible to amoxicillin/clavulanic acid (MIC 0.094), piperacillin/tazobactam (MIC 0.75), imipenem (MIC 0.023), and clindamycin (MIC 1), as well as metronidazole (MIC 1). However, it was resistant to penicillin (MIC 12). The results were interpreted using EUCAST (European Committee on Antimicrobial Susceptibility Testing) breakpoints for *Bacteroides* spp. [1].

## 4 Conclusion and Results

The patient continued treatment with piperacillin/tazobactam, while vancomycin was discontinued at day seven of hospitalization when the microorganism was identified, and culture results ruled out the involvement of Gram‐positive bacteria. This treatment was administered for a total of 7 days to cover a broad spectrum of microorganisms. The patient did not experience any adverse effects. After 1 week, owing to clinical amelioration, the therapeutic regimen was changed to oral levofloxacin in conjunction with metronidazole to also treat *P. aeruginosa* isolated in the urine culture. Following positive clinical progress, the patient was discharged home 12 days after admission.

The patient continued antibiotic treatment for 8 weeks. However, due to gastrointestinal side effects, the medication was changed to moxifloxacin. At Week 8, the patient experienced the adverse effect of neutropenia, prompting a switch to clindamycin for 14 weeks, which was well tolerated. In total, the patient received 28 weeks of antibiotic treatment, resulting in good clinical improvement without recurrence of infection. Subsequent control examinations, including scintigraphy with labeled leukocytes, did not show any images suggestive of periprosthetic infection after 7 months of treatment. Currently, the patient is in good health and is receiving regular follow‐up care from the hospital's infectious disease service.

## 5 Discussion

The implantation of joint prostheses has been one of the great advances in orthopedic surgery in recent decades, improving the quality of life of many patients. However, complications can arise, including infection [2].

Septic or infectious arthritis is the invasion of the joint space by various microorganisms, including bacteria, viruses, and fungi, directly or (more frequently) indirectly through the hematogenous route. Although any infectious agent can cause septic arthritis, the most common are gram‐positive cocci (staphylococci, streptococci, enterococci) and gram‐negative bacilli. Anaerobes are considered rare causative agents of septic arthritis, with *Cutibacterium acnes* being the most frequent [2].

Anaerobes constitute components of the microbiota within the gastrointestinal and genitourinary tracts. Disturbances at these anatomical sites, arising from diverse scenarios (infectious processes, surgical interventions), may result in the dissemination of these bacteria and their migration into sterile cavities, thereby causing infectious processes [3].

*B. fragilis* is an anaerobic, non‐spore‐forming, bile‐resistant, Gram‐negative bacillus that forms part of the human intestinal microbiota. It belongs to the *Bacteroides* genus of the Bacteroidaceae family in the Bacteroidales order [3]. The first species of the genus *Bacteroides* was described in 1898 as *Bacillus fragilis* and reclassified as *B. fragilis* in 1919 [4]. In recent decades, the taxonomy of the genus has undergone various modifications. Currently, the following species are grouped within the *B. fragilis* group: *B. acidifaciens* , *B. caccae* , *B. cellulosilyticus* , *B. eggerthii* , *B. faecis* , *B. fragilis division I*, *B. fragilis division II*, *B. helcogenes* , *B. intestinalis* , *B. nordii* , *B. ovatus* , *B. pyogenes* , *B. salyersiae* , *B. stercoris* , *B. thetaiotaomicron* , *B. uniformis* , and *B. xylanisolvens* . Additionally, there has been a reclassification of *Bacteroides distasonis* , *Bacteroides merdae*, and *Bacteroides goldsteinii* in a new genus, *Parabacteroides*. Notably, *B. vulgatus* is now known as *Phocaeicola vulgatus* [5, 6].

The genus *Bacteroides*, like the vast majority of anaerobes, is frequently associated with polymicrobial infections, mainly intra‐abdominal [4]. Different species of *Bacteroides*, especially *B. fragilis* , are often isolated in intra‐abdominal infections, including intraperitoneal and visceral abscesses, as well as peritonitis associated with conditions such as diverticulitis, appendicitis, and intestinal anastomosis. They are also commonly isolated in cases of long‐bone osteomyelitis, especially after trauma or fracture, and in infections related to peripheral vascular disease, pressure ulcers, cranial and facial bone osteomyelitis, and pelvic osteomyelitis after surgery. *B. fragilis* has also been implicated in surgical wound infections and abscesses, although it is rarely described as a causative agent in prosthetic infections [7, 8, 9, 10, 11, 12].

To our knowledge, this is the first reported case of a *B. fragilis* infection in a megaprosthesis in a patient with Ewing's sarcoma. This unique case involves an oncology patient who did not present with gastrointestinal symptoms, had no comorbidities, and had a megaprosthesis. Septic arthritis due to this microorganism has been described in both native and prosthetic joints associated with abdominal infections, as well as in patients with chronic diseases such as rheumatoid arthritis [13] or other comorbidities such as alcohol consumption [8, 9]. Typically, the focus of infection is the gastrointestinal tract and skin lesions [10].

The challenges associated with etiological diagnosis call for proper sample collection, transport, conservation, and culture techniques. The introduction of new diagnostic methods in microbiology laboratories, such as MALDI‐TOF MS, has enabled more reliable and faster identification, leading to the establishment of more appropriate treatments. A few recent studies substantiate the precision of MALDI‐TOF MS for the identification of *B. fragilis* group (BFG) species [6, 14, 15], underscoring the clinical utility of this analytical technique.

The susceptibility of *B. fragilis* was found to be variable by Aldridge [16], depending on the infection location. Each antimicrobial agent exhibited comparable activity against the microorganism, although isolates from body fluids tended to be slightly more resistant to ceftizoxime, ceftriaxone, and cefmetazole compared to those from abscesses and wounds. Piperacillin–tazobactam, ticarcillin–clavulanate, and ampicillin–sulbactam demonstrated good activity. However, all study isolates were resistant to penicillin G due to beta‐lactamase production. No resistance to metronidazole or imipenem was detected among strains isolated from blood [16, 17]. Nevertheless, multiple reports of resistance to metronidazole and the use of imipenem have been described recently [5]. Increasing rates of resistance have also been reported to clindamycin and cefoxitin, suggesting limited utility in the absence of susceptibility testing to the isolate in question [5, 18].

According to the clinical practice guidelines of the Infectious Diseases Society of America, the recommended medical treatment for a patient with a prosthetic joint infection after debridement and retention of the prosthesis is 4–6 weeks of high‐quality oral or intravenous antimicrobial therapy with specific bioavailability for the pathogen [19]. In the case presented here, the duration of antibiotic treatment was prolonged due to the various side effects induced by the different antibiotics administered to the patient. Also playing a role in this decision was the unfavorable prognosis associated with the specific tumor prosthesis, together with limited clinical experience and the absence of consensus on the optimal duration of antibiotic use.

In a retrospective study analyzing the treatment of megaprosthesis infections in patients with bone and soft tissue tumors over an 8‐year period, 28 patients were studied, including five cases of proximal femoral prosthesis infections [20]. The perioperative and postoperative antibiotic protocol included broad‐spectrum empirical coverage with a third‐generation cephalosporin (cefoperazone) and vancomycin or teicoplanin for MRSA infection. Subsequently, the infectious diseases unit modified the antibiotic therapy based on culture results and antibiotic susceptibility.

All patients except one had their infection completely resolved. The most frequently involved pathogens were *Staphylococcus* spp. (methicillin‐susceptible *Staphylococcus aureus* [MSSA], coagulase‐negative *Staphylococcus* [CONS], methicillin‐resistant *S. aureus* [MRSA], and *Staphylococcus epidermidis* ), which were present in 18 patients, with three cases being polymicrobial. The median duration of antimicrobial therapy was 6 weeks (1–12 weeks). In our case, unlike the mentioned study, *B. fragilis* was isolated in pure culture and the patient received 28 weeks of antibiotic treatment. The precise way in which the patient acquired the infection remains uncertain, as she did not report any gastrointestinal symptoms. Her medical history of interest included a neoplastic intervention and residency in a long‐term care facility. It is also unclear whether the neoplasm contributed to the acquisition of the infection, as the patient was not immunocompromised. To the best of our knowledge, there is no documented evidence in the literature establishing an association between a history of this malignant tumor, prolonged stays in a care facility, and an increased risk of *B. fragilis* infection.

## 6 Conclusion

In this report, we present the first documented case of a prosthetic infection (megaprosthesis) associated with *B. fragilis* in a patient diagnosed with Ewing's Sarcoma. The patient underwent a treatment regimen involving debridement and an extended course of combination antibiotic therapy spanning 7 months. This therapeutic approach resulted in the preservation of the megaprosthesis and culminated in a complete recovery. Further investigations and a protracted follow‐up are deemed essential to foster a more comprehensive understanding of the treatment and long‐term outcomes in similar cases. Additionally, this case underscores the importance of research to elucidate the emergence of unusual pathogens in unforeseen clinical contexts.

## Author Contributions

**Yoleidys Martínez‐Ysasis:** conceptualization, investigation, writing – original draft, writing – review and editing. **Antonella Francesca Simonetti:** investigation, supervision, validation, visualization, writing – original draft, writing – review and editing. **Ferran Navarro:** supervision, validation, visualization.

## Ethics Statement

Ethical approval was granted by the Hospital de la Santa Creu i Sant Pau Research Ethics Committee.

## Consent

Written informed consent was obtained from the patient to publish this report in accordance with the journal's patient consent policy.

## Conflicts of Interest

The authors declare no conflicts of interest.

## Data Availability

The data that support the findings of this study are available from the corresponding author upon reasonable request.
